# Bimatoprost for Eyelash Growth in Japanese Subjects: Two Multicenter Controlled Studies

**DOI:** 10.1007/s00266-014-0293-7

**Published:** 2014-03-19

**Authors:** K. Harii, S. Arase, R. Tsuboi, E. Weng, S. Daniels, A. VanDenburgh

**Affiliations:** 1 Department of Plastic Surgery, Kyorin University School of Medicine, Tokyo, Japan; 2 Health Insurance Naruto Hospital, Tokushima, Japan; 3 Department of Dermatology, Tokyo Medical University, Tokyo, Japan; 4 Dermatology Clinical Research and Development, Allergan, Inc., Irvine, CA USA; 5 Global Health Outcomes, Strategy and Research, Allergan, Inc., Irvine, CA USA; 6 Dermatology Clinical Research and Development, Allergan, Inc., 2525 Dupont Drive, Mail Stop T1-2N, Irvine, CA 92612 USA

**Keywords:** Bimatoprost, Chemotherapy, Eyelashes, Hypotrichosis, Japanese, Patient satisfaction

## Abstract

**Background:**

Bimatoprost 0.03 % has enhanced eyelash prominence in clinical trials enrolling mostly Caucasian subjects. The studies described in this report evaluated the efficacy and safety of bimatoprost in Japanese subjects with idiopathic and chemotherapy-induced eyelash hypotrichosis.

**Methods:**

In two multicenter, double-masked, randomized, parallel-group studies (study 1: *n* = 173 [idiopathic]; study 2: *n* = 36 [chemotherapy-induced]), subjects received bimatoprost 0.03 % or vehicle applied once daily to the upper eyelid margins. The primary efficacy measure was eyelash prominence measured by Global Eyelash Assessment (GEA) scores. Additional measures were eyelash length, thickness, and darkness, assessed by digital image analysis, and patient satisfaction (Eyelash Satisfaction Questionnaire-9). Safety assessments included adverse-event monitoring and ophthalmic examinations.

**Results:**

Significantly more bimatoprost-treated subjects had at least a one-grade improvement in GEA score from baseline to month 4 compared with vehicle in study 1 (77.3 vs 17.6 %; *P* < 0.001) and study 2 (88.9 vs 27.8 %; *P* < 0.001). Bimatoprost-treated subjects had significantly greater increases in eyelash length, thickness, and darkness at the primary time point (month 4 in both studies; all *P* < 0.001, study 1; *P* ≤ 0.04, study 2). The bimatoprost group showed greater subject satisfaction in both studies. The incidence of adverse events was similar in the two groups. Ophthalmic examination showed slightly greater mean reductions in intraocular pressure (IOP) with bimatoprost than with vehicle, and the reductions were within the normal range for daily IOP fluctuations.

**Conclusion:**

Bimatoprost 0.03 % was shown to be effective and safe in these studies of Japanese subjects with eyelash hypotrichosis.

**Level of Evidence I:**

This journal requires that authors assign a level of evidence to each article. For a full description of these Evidence-Based Medicine ratings, please refer to the Table of Contents or the online Instructions to Authors www.springer.com/00266.

## Introduction

Eyelashes are important in the concept of physical beauty, and many women consequently use cosmetic approaches to obtain longer and thicker lashes [1–3]. Mascara is frequently used to darken, thicken, and lengthen existing eyelashes [1, 4]. In addition, a variety of over-the-counter products containing natural extracts, vitamins, prostaglandin analogs, or proprietary peptides are claimed to enhance eyelashes, but they have not been well studied, and their effectiveness remains unproven [4].

Individuals seek eyelash enhancements for a variety of reasons. Besides aesthetic reasons, some individuals exhibit inadequate or sparse eyelashes (i.e., hypotrichosis) characterized by reduced hair growth, which may be attributed to known etiologies such as age, hereditary factors, chemotherapy, and other medical treatments, but often the etiology is unknown [5].

Individuals affected by alopecia and other associated conditions of hair loss that might include loss of eyelashes and eyebrows often have issues with self-identity because they lack key facial features [6]. Hair loss, including loss of eyelashes, is common among patients undergoing chemotherapy and is considered a troubling adverse effect that can have psychosocial consequences [7, 8].

Bimatoprost 0.03 % has been developed for treating hypotrichosis of the eyelashes. This synthetic prostaglandin structural analog, known as a prostamide, is the same active ingredient at the same concentration (0.03 %) found in bimatoprost ophthalmic solution used for treatment of open-angle glaucoma and ocular hypertension [9–11].

Eyelash growth was noted as an adverse effect of bimatoprost use in clinical trials of patients with glaucoma and ocular hypertension [9, 10, 12, 13], an effect thought to arise by the interaction of bimatoprost with prostamide-sensitive receptors in hair follicles [14].

Bimatoprost 0.03 % solution has been shown in several clinical trials to enhance eyelash growth when applied topically to the upper eyelid margins [15–18]. On the basis of these findings, approval of bimatoprost 0.03 % for treating hypotrichosis of the eyelashes was granted on several continents, including North America and parts of Asia.

The studies published to date were conducted primarily with Caucasian subjects [15, 18]. However, differences have been observed between Asian and Caucasian individuals in the number, thickness, and curliness of eyelashes [19]. Therefore, the two clinical trials described in this report aimed to evaluate the efficacy and safety of bimatoprost 0.03 % for Japanese subjects with hypotrichosis of the eyelashes.

## Methods

### Study Design and Subjects

The reported multicenter, double-masked, randomized, parallel-group, vehicle-controlled studies had a similar design and a duration of 5 months. Study 1 was conducted at eight centers in Japan with subjects who had idiopathic hypotrichosis of the eyelashes, and study 2 was conducted at nine centers in Japan with subjects who had chemotherapy-induced hypotrichosis of the eyelashes. Three centers participated in both studies.

The trial period of each study ran from July 2011 to May 2012. Each study was performed in compliance with Japanese Good Clinical Practice, Good Clinical Practice, and the International Conference on Harmonisation guidelines. Institutional review board approval was obtained at each site before initiation of the study, and all the subjects gave written informed consent.

In both studies, subjects 20 years of age or older were eligible if they had a Global Eyelash Assessment (GEA) score of 1 or 2 using the GEA with the associated Photonumeric Guide for Japan (GEA-J), a best-corrected visual acuity score of 20/100 or better for each eye, and an intraocular pressure (IOP) of 20 mmHg or lower in each eye. In study 2, the eligible subjects also were required to report chemotherapy-induced eyelash hypotrichosis after completion of standard adjuvant chemotherapy for an early-stage solid tumor. The chemotherapy had to be completed 4–24 weeks before baseline assessments, and all side effects related to the chemotherapy had to be grade 1 or less on the Common Terminology Criteria for Adverse Events (CTCAE, version 4.03). Subjects were excluded from both studies if they had any uncontrolled systemic disease, any known ocular disease or abnormality, or a history of ocular surgery within the previous 3 months.

The subjects were screened for eligibility 3–14 days before randomization. The eligible subjects were randomly assigned in a 1:1 ratio to bimatoprost 0.03 % or vehicle by an interactive voice or by a Web response system on day 1 (baseline). The study medication was dosed once nightly for 4 months. One drop of study medication was applied to a sterile, single-use applicator and brushed along the upper eyelid margins. Separate applicators were used for each eye. The subjects were advised to remove makeup and contact lenses before application of the study medication. Contact lenses could be reinserted after 30 min. The subjects returned to the study center for evaluation at the end of week 1, then at months 1, 2, 4 (end of treatment), and 5 (posttreatment follow-up assessment).

### Efficacy Assessments

The primary efficacy measurement was overall eyelash prominence as determined by the GEA score using the GEA-J. The GEA-J scale is identical to the original GEA scale except that the associated photoguide contains photographs of Japanese subjects exclusively. Both the GEA and the GEA-J scales have been validated in previous clinical studies [15]. A GEA score of 1 indicates none to minimal eyelash prominence; a score of 2 indicates moderate eyelash prominence; a score of 3 indicates marked eyelash prominence; and a score of 4 indicates very marked eyelash prominence.

The investigators assigned GEA scores based on live, in-person assessments of overall eyelash prominence across both eyes. The secondary efficacy end points included measurements of upper eyelash length (mm), eyelash thickness (mm^2^), and eyelash darkness (intensity units) using a validated digital image analysis (DIA) of superior-view digital eyelash photographs taken with standardized equipment and subject positioning at each study visit (except for week 1). Patient-reported satisfaction with various attributes of eyelashes also was assessed at each visit except for week 1 using the 9-item Eyelash Satisfaction Questionnaire (ESQ-9).

The ESQ-9 consists of nine questions and three domains. Each question is measured on a 5-point Likert-type scale (i.e., very satisfied, satisfied, neutral, unsatisfied, very unsatisfied), and each domain consists of three questions. The three domains are Length, Fullness, and Overall Satisfaction (LFOS: domain 1, assessing subject satisfaction with physical attributes of eyelashes); Confidence, Attractiveness, and Professionalism (CAP: domain 2, assessing subject satisfaction with subjective attributes of eyelash health); and Daily Routine (DR: domain 3, assessing subjects’ perceptions of the degree of bother associated with making eyelashes presentable). Positive changes in ESQ-9 scores and domains indicate improvement. The ESQ-9 has been translated, culturally adapted, and psychometrically validated with the Japanese population.

### Safety

Ophthalmic examinations were performed at screening and at months 1 and 4. The exams included biomicroscopy and dilated ophthalmoscopy as well as measurement of best-corrected visual acuity and IOP. Adverse events (AEs) were recorded at each visit. Physical examinations and clinical laboratory testing were performed at screening and at month 4, and vital signs were obtained at each visit except for week 1.

### Statistical Analyses

The sample size determination in study 1 indicated that a total enrollment of 126 subjects would provide 90 % statistical power for detecting a 30 % difference in the response rate between the treatment groups, assuming a 30 % response in the vehicle control group and a two-sided type 1 error of 0.05. Study 2 was primarily a safety study not powered for efficacy and enrolled a much smaller cohort.

All efficacy analyses were conducted on the intent-to-treat (ITT) population, which comprised all of the randomized subjects whether they had received study treatment or not. The primary efficacy end point was prospectively identified as the clinical response and defined as at least a one-grade increase in GEA score from baseline at month 4. Between-group comparisons of the response rate were made using Pearson’s Chi square test or Fisher’s exact test. The same method was used to compare the response rates between the treatment groups at months 1, 2, and 5.

The secondary efficacy end points of change from baseline to month 4 (the primary time point) in eyelash length, progressive eyelash thickness, and eyelash darkness on DIA were calculated as the average of right and left upper eyelash measurements. Between-group comparisons were performed using the Wilcoxon rank-sum test. The ESQ-9 scores for each question and domain (three domains consisting of three questions each) were analyzed. The change from baseline on each question was analyzed by descriptive statistics.

Between-group comparisons for each domain were made using the Wilcoxon rank-sum test. Missing efficacy data were imputed using a last-observation-carried-forward approach up to month 4. No data imputation was made for missing month 5 data. Differences were considered statistically significant if the two-sided *P* value was 0.05 or lower.

Correlation analyses of the change from baseline to month 4 in GEA versus ESQ-9 question 3 (“Overall, how satisfied are you with your eyelashes?”), domain 1, and domain 2 were performed to evaluate the direction and strength of the correlation using the Spearman rank correlation coefficient (*ρ*). Its significance was assessed by the *t* test.

Safety parameters were evaluated in the safety population, which consisted of all the subjects who received at least one dose of study medication. The AEs were coded using the Medical Dictionary for Regulatory Activities, version 15.0. Comparisons of safety parameters were made using appropriate nonparametric statistical methods (Fisher’s exact test, Pearson’s Chi square test, or Wilcoxon rank sum test) or parametric tests (analysis of variance). No imputation of missing safety data was made.

## Results

### Subject Disposition and Demographics

For study 1, a total of 173 subjects were randomized: 88 subjects to bimatoprost and 85 subjects to vehicle. The two treatment arms were comparable in terms of baseline characteristics. The overall mean age was 40.8 years, and 89.6 % of the subjects were women. A majority of the patients (69.9 %) had a baseline GEA score of 2 (Table 1). One subject with a baseline GEA score of 3 was randomized to the bimatoprost group but did not receive treatment. This subject was included in the efficacy analyses as part of the ITT population.

**Table 1:** Subject demographics and baseline characteristics

	Study 1		Study 2	
	Bimatoprost 0.03 %	Vehicle	Bimatoprost 0.03 %	Vehicle
	(*n* = 88)	(*n* = 85)	(*n* = 18)	(*n* = 18)
	*n* (%)	*n* (%)	*n* (%)	*n* (%)
Mean age: years (range)	40.1 (20–68)	41.4 (20–64)	46.3 (31–61)	54.9 (39–74)
Age distribution				
<45 years	58 (65.9)	48 (56.5)	8 (44.4)	3 (16.7)
45–65 years	29 (33.0)	37 (43.5)	10 (55.6)	12 (66.7)
>65 years	1 (1.1)	0 (0)	0 (0)	3 (16.7)
Gender				
Males	9 (10.2)	9 (10.6)	0 (0)	0 (0)
Females	79 (89.8)	76 (89.4)	18 (100)	18 (100)
Baseline GEA score				
1 (minimal)	30 (34.1)	21 (24.7)	12 (66.7)	14 (77.8)
2 (moderate)	57 (64.8)	64 (75.3)	6 (33.3)	4 (22.2)
3 (marked)	1 (1.1)^a^	0 (0)	0 (0)	0 (0)

*GEA* Global Eyelash Assessment

All the subjects were Japanese

^a^The subject was randomized but did not receive treatment

Study 2 randomized 36 subjects to treatment, with 18 subjects in each group. The groups were comparable except for age. All of the subjects were women, and the majority (72.2 %) had a GEA score of 1 at baseline (Table 1).

### Efficacy: GEA Scores

**Fig. 1:**
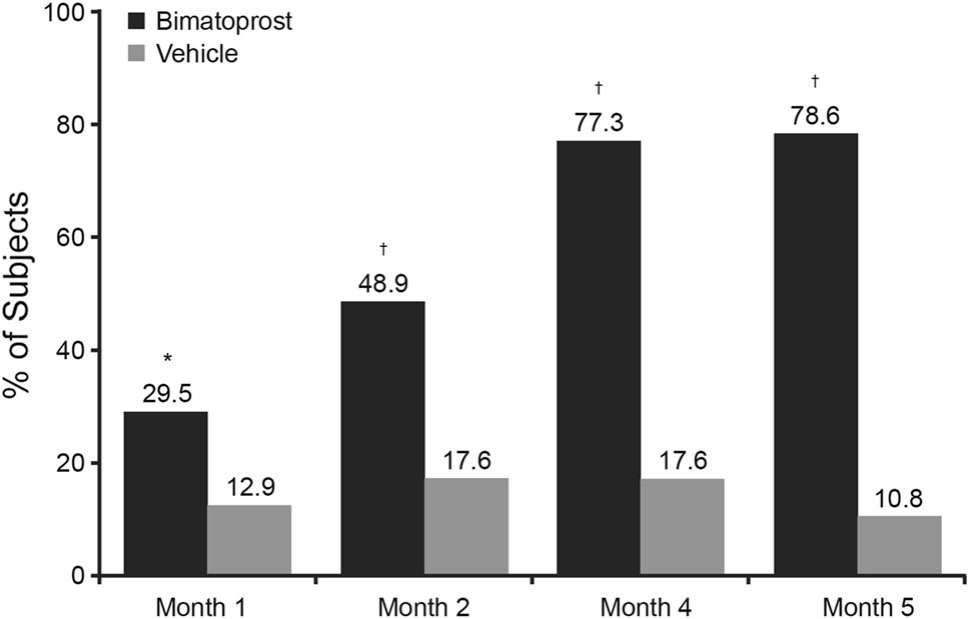
Percentage of subjects in study 1 with at least a one-grade improvement in Global Eyelash Assessment score. The study treatment was applied once nightly for 4 months. The subjects did not use the study treatment between months 4 and 5. *P = 0.008. ^†^ *P* < 0.001 versus vehicle

The proportion of subjects who responded to treatment with at least a one-grade improvement in GEA score from baseline to month 4 was significantly greater with bimatoprost than with vehicle in both study 1 (77.3 vs 17.6 %; *P* < 0.001) and study 2 (88.9 vs 27.8 %; *P* < 0.001). In study 1, significantly higher response rates with bimatoprost, reflecting improved eyelash prominence, were evident by month 1 and maintained at the month 5 posttreatment assessment (Fig. 1). In addition, a significantly greater proportion of patients in the bimatoprost group than in the vehicle group experienced at least a two-grade improvement in GEA score from baseline, which started at month 2 and continued through the posttreatment assessment at month 5 (38.1 vs 1.2 % at month 5; *P* < 0.001).

**Fig. 2:**
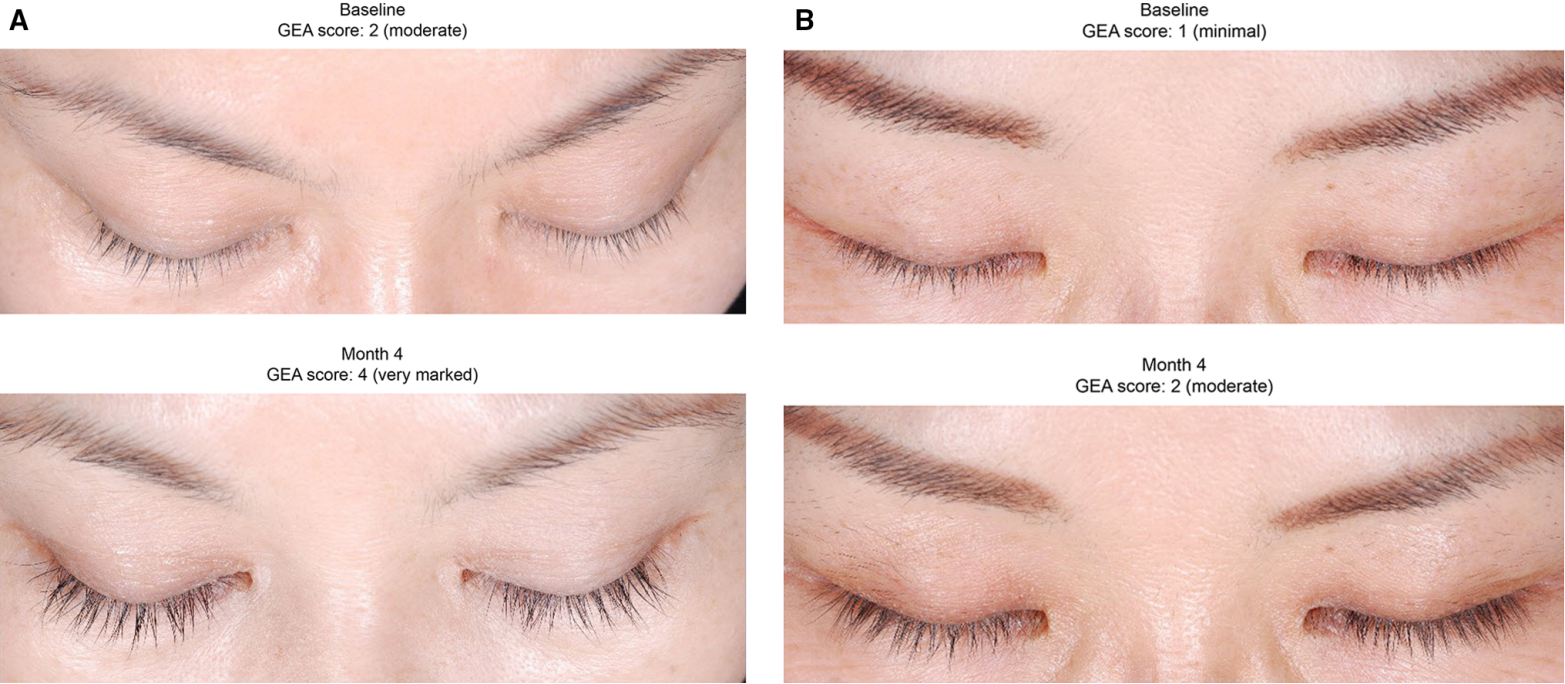
Example of subjects from **a** study 1 and **b** study 2 who responded to bimatoprost treatment with two- and one-grade improvements, respectively, in Global Eyelash Assessment (GEA) score at month 4

Although study 2 was not powered for efficacy, the statistically significant between-group difference in eyelash prominence achieved at the month 4 primary time point was maintained at the month 5 posttreatment assessment (83.3 vs 27.8 %; *P* < 0.001). An example of a subject with a two-grade improvement at month 4 in study 1 and a one-grade improvement at month 4 in study 2 is shown in Fig. 2.

### Efficacy: DIA

**Fig. 3:**
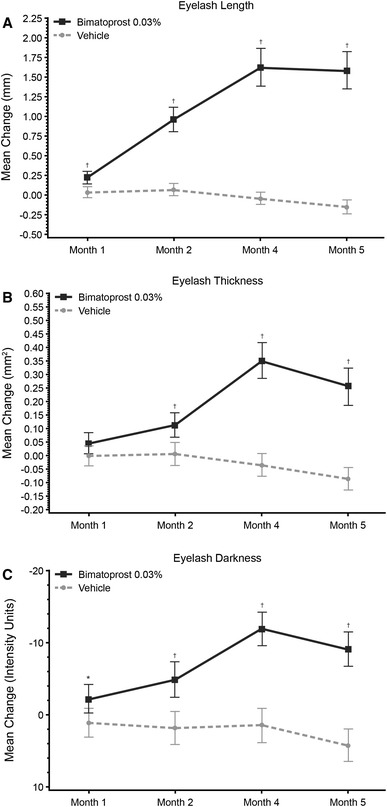
Mean changes from baseline (± 2 times standard error) in **a** upper eyelash length, **b** thickness, and darkness in study 1. The study treatment was applied once nightly for 4 months. The subjects did not use the study treatment between months 4 and 5. A negative change in eyelash darkness indicates darkening. *P = 0.012. ^†^ *P* < 0.001 versus vehicle

In both studies, the bimatoprost-treated subjects experienced significantly greater improvements than the vehicle group from baseline to month 4 in upper eyelash length, thickness, and darkness. In study 1, from baseline to month 4, the bimatoprost-treated subjects experienced mean improvements of 1.62 mm (24 %) in upper eyelash length, 0.35 mm^2^ (44.8 %) in upper eyelash thickness, and −12.02 intensity units (negative value represents darkening; 8 %) in upper eyelash darkness (Fig. 3). In contrast, these eyelash parameters were essentially unchanged in the vehicle group.

**Fig. 4:**
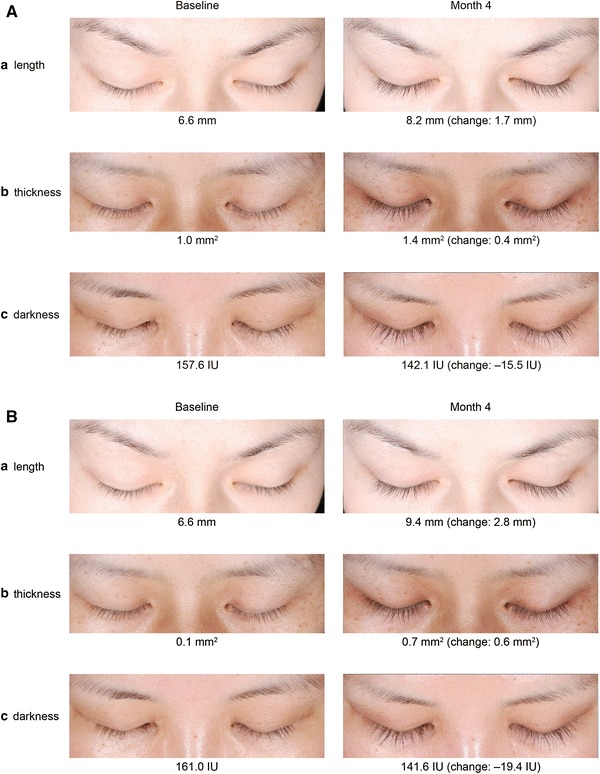
Examples of subjects who responded to bimatoprost treatment with an increase in **a** upper eyelash length, **b** thickness, and **c** darkness, including baseline and month 4, as well as a change from baseline values (measurements are rounded to the nearest whole number). The individual changes in these subjects were comparable with the mean changes observed in the bimatoprost group in **a** study 1 and **b** study 2. *IU* intensity units

Significant between-group differences were observed for mean change in eyelash length and darkness, starting at month 1, and for mean change in eyelash thickness, starting at month 2. These differences were maintained through month 5 (all *P* < 0.001 except for eyelash darkness at month 1 [*P* = 0.012]). In study 2, improvements in these parameters with bimatoprost compared with vehicle were statistically significant at month 4 (mean change from baseline in eyelash length [2.65 vs 0.99 mm; *P* = 0.003], eyelash thickness [0.76 vs 0.28 mm^2^; *P* = 0.007], and eyelash darkness [−21.07 vs −4.60 intensity units; *P* = 0.040). Examples of improvements in eyelash length, thickness, and darkness for bimatoprost-treated subjects in both studies are shown in Fig. 4.

### Patient-Reported Satisfaction: ESQ-9

In both studies, both treatment groups had similar mean baseline scores for the nine questions and three domains of the ESQ-9. In study 1, the bimatoprost group had significantly greater improvements from baseline satisfaction scores in domains 1 (LFOS) and 2 (CAP) (both *P* ≤ 0.001) at multiple time points and in the individual questions comprising these domains than the vehicle group (*P* ≤ 0.028). For question 3 in domain 1 (“Overall, how satisfied are you with your eyelashes?”), the bimatoprost group reported significantly greater improvements in satisfaction scores from baseline than the vehicle group at months 2–5 (*P* < 0.001) (Table 2).

**Table 2:** Significant differences on the 9-item Eyelash Satisfaction Questionnaire (ESQ-9) by time period between the bimatoprost and vehicle groups

Item	Question	Study 1		Study 2	
		Time period significant^a^	*P* value	Time period significant^a^	*P* value
1	Length satisfaction	Months 2–5	<0.001	Months 2–5	≤0.006
2	Fullness/thickness satisfaction	Months 2–5	≤0.002	Months 2–5	≤0.035
3	Overall satisfaction	Months 2–5	<0.001	Months 4–5	≤0.006
4	Time applying mascara	None	≥0.335	None	≥0.312
5	Time removing mascara	None	≥0.058	None	≥0.295
6	Hassle with eyelashes	None	≥0.070	None	≥0.189
7	Feel confident about looks^b^	Months 2–5	≤0.028	Month 4	0.044
8	Feel confident in appearance^b^	Months 4–5	≤0.007	Month 4	0.014
9	Feel attractive^b^	Months 4–5	≤0.004	Months 2–4	≤0.050
LFOS	Domain 1 (questions 1–3)	Months 2–5	≤0.001	Months 2–5	≤0.017
CAP	Domain 2 (questions 7–9)	Months 4–5	<0.001	Month 4	0.019
DR	Domain 3 (questions 4–6)	None	≥0.317	None	≥0.123

*LFOS* length, fullness, and overall satisfaction, *CAP* confidence, attractiveness, and professionalism, *DR* daily routine; *ESQ-9* nine-item Eyelash Satisfaction Questionnaire

^a^Assessments were made at baseline and at months 1, 2, 4, and 5. Time period column shows the months at which significant differences favoring bimatoprost versus vehicle were observed

^b^Questions were asked in the context of appearance without the use of mascara

No significant between-group differences were observed for domain 3 (DR) or its individual questions. At month 4, for the bimatoprost group, the improvements from baseline in subject satisfaction with eyelashes, as measured by question 3 (*ρ* = 0.381), domain 1 (*ρ* = 0.451), and domain 2 (*ρ* = 0.409), were positively and significantly correlated with improvements from baseline in eyelash prominence, as measured by GEA score (all *P* < 0.001). Significant correlations were not seen in the vehicle group.

In study 2, compared with the vehicle group, the bimatoprost group also had significantly greater improvements from baseline in satisfaction scores at multiple time points in domains 1 (*P* ≤ 0.017) and 2 (*P* = 0.019) and in the individual questions comprising these domains (*P* ≤ 0.05). Again, in the bimatoprost group, subject satisfaction with eyelashes, as measured by question 3 (*ρ* = 0.641; *P* = 0.004), domain 1 (*ρ* = 0.516; *P* = 0.028), and domain 2 (*ρ* = 0.538; *P* = 0.021) in ESQ-9, were significantly correlated with improvements in eyelash prominence measured by the GEA score.

### Safety Profile: AEs

In study 1, the overall incidence of AEs during the entire study was similar in the bimatoprost and vehicle groups (41.4 vs 37.6 %; *P* = 0.643). The most common AEs in the bimatoprost group were nasopharyngitis (13.8 %), conjunctival hyperemia (4.6 %), punctate keratitis (3.4 %), eye discharge (3.4 %), and skin hyperpigmentation (3.4 %). None occurred at a significantly higher rate than in the vehicle group. Each of these common AEs was reported as mild in severity.

Overall, most AEs resolved before the end of the study, and the vast majority were of mild severity. None of the AEs reported as moderate or severe was considered by the investigator to be associated with the study treatment. The serious AEs in the bimatoprost group included one case each of appendicitis and pyelonephritis, which were not considered to be associated with treatment. Three subjects discontinued the study due to AEs, namely, dry eye and facial pain (each considered to be treatment related) in the bimatoprost group and dissociative disorder in the vehicle group.

In study 2, the overall incidence of AEs in the bimatoprost group during the entire study was 66.7 % compared with 72.2 % in the vehicle group (*P* > 0.999). The types of AEs did not differ significantly between the two groups. Radiation skin injury (related to previous cancer treatment) and skin hyperpigmentation were the most common AEs in the bimatoprost group, whereas nasopharyngitis, radiation skin injury (related to previous cancer treatment), and insomnia were the most common AEs in the vehicle group. No other AEs were reported for more than one subject in either group. No subjects in the bimatoprost group had serious AEs, and no subjects discontinued the study due to AEs.

### Safety Profile: Ophthalmic Examination

In study 1, ophthalmic examination showed no statistically or clinically significant differences between the bimatoprost and vehicle groups in terms of change from baseline in IOP (mean change from baseline to month 1, −0.79 vs −0.47 mmHg, *P* = 0.264; mean change from baseline to month 4, −0.84 vs −0.23 mmHg, *P* = 0.085). No subject had an IOP of 6 mmHg or lower, and no subject had an AE report of reduced IOP. Visual acuity remained unchanged (between −2 and +2 lines) for more than 90 % of the patients in both groups (*P* = 0.248) at month 1 and for more than 85 % at month 4 (*P* = 0.257). Biomicroscopy and ophthalmoscopy findings of a one-grade increase from baseline were observed more frequently in the bimatoprost group and reflected higher rates of conjunctival hyperemia (9.2 vs 0 %; *P* = 0.007). No two-grade increases were found.

In study 2, the bimatoprost group had a statistically significantly greater reduction in IOP from baseline than the vehicle group at the month 1 time point only (−2.21 vs −0.68 mmHg; *P* = 0.007). These changes in IOP were not considered clinically meaningful and, at month 4, the changes in IOP did not differ significantly between the treatment groups (−1.50 vs −0.38 mmHg; *P* = 0.152). As in study 1, no subject had an IOP of 6 mmHg or lower, and no subject had an AE report of reduced IOP. Visual acuity remained unchanged in most of the subjects, except for a decline of more than two lines in one subject and an increase of more than two lines in one subject, both in the bimatoprost group. The biomicroscopy and ophthalmoscopy findings did not differ between the treatment groups. No clinically meaningful changes in vital signs, hematology, blood chemistry, or urinalysis laboratory data were observed during either study.

## Discussion

These randomized clinical trials demonstrated that bimatoprost 0.03 % applied to upper eyelid margins once nightly for 4 months was effective and safe for Japanese subjects with hypotrichosis of the eyelashes, regardless of etiology. A significantly greater proportion of the bimatoprost-treated subjects experienced improved eyelash prominence than the vehicle-treated subjects, as measured by the GEA score. The bimatoprost-treated subjects also had significantly greater improvements in upper eyelash length, thickness, and darkness, as measured by DIA, and greater improvements in satisfaction with their eyelashes, as measured by ESQ-9, than the subjects in the vehicle control group.

In study 1, bimatoprost produced significant effects on eyelash prominence, length, and darkness, starting at month 1, and on eyelash thickness, starting at month 2. The bimatoprost-treated subjects in study 1 also experienced significantly better satisfaction compared with baseline by month 2 than the vehicle-treated subjects in terms of length, fullness, and overall satisfaction with their eyelashes. Increased improvements in these end points were seen until the final on-treatment assessment at month 4 (the primary time point), and the improvements were maintained in the posttreatment assessment at month 5. Thus, bimatoprost produced robust and sustained eyelash improvements in Japanese subjects with hypotrichosis of the eyelashes.

Study 2 had a smaller sample but exhibited similar temporal trends for efficacy, with significant effects in the bimatoprost group compared with the vehicle group at month 4 (the primary time point) on the end points of eyelash prominence, length, thickness, darkness, and overall satisfaction and at month 2 on the end points of satisfaction with eyelash length and fullness. These effects were maintained at the post-treatment assessment at month 5 for most end points.

Although some differences have been noted in the eyelash morphology of Asians compared with Caucasians (e.g., number, thickness, and curliness of eyelashes) [19], the proportion of bimatoprost-treated subjects with at least a one-grade improvement in GEA score from baseline to month 4 was comparable between the Japanese subjects in study 1 and a mostly Caucasian, North American cohort with idiopathic hypotrichosis of the eyelashes evaluated in a prior study (77.3 and 78.1 %, respectively) [15]. In both populations, the improvement in eyelash prominence was maintained at the post-treatment assessment at month 5. Moreover, in both populations, the GEA score improvements were significantly correlated with greater patient-reported satisfaction with eyelashes, as measured with ESQ-9 in the current study and with the original 23-item ESQ in the North American study [15].

The safety profile of bimatoprost 0.03 % has been well established for treatment of open-angle glaucoma and ocular hypertension [11, 20]. Although the same 0.03 % drug concentration was used for treatment of hypotrichosis of the eyelashes, the total dose applied to the eyelid margins was only approximately 5 % of the dose compared with an eyedrop [21]. The types of AEs reported in the current trials were consistent with those of prior studies investigating mostly Caucasian populations with hypotrichosis of the eyelashes [15, 17]. The overall incidence of AEs and that of each AE type did not differ significantly between the two treatment groups.

The ophthalmic examinations conducted at months 1 and 4 further confirmed the safety of bimatoprost 0.03 % for treatment of hypotrichosis of the eyelashes and indicated a good safety profile in Japanese subjects with hypotrichosis. In both studies, small mean reductions from baseline IOP were observed in the bimatoprost and vehicle groups but were not clinically significant.

In study 2, the bimatoprost group had a slightly greater mean reduction in IOP from baseline at month 1 than the vehicle group, which was statistically significant. However, no clinically meaningful change in IOP occurred in any subject, and no subject had an IOP of 6 mmHg or less at either on-treatment examination. The normal range for IOPs is reportedly 7–21 mmHg, and daily fluctuations in IOP can show an approximate range of 3–7 mmHg [22–25]. Accordingly, the changes observed in the current studies were well within normal ranges, consistent with the low exposure to bimatoprost after topical application to the upper eyelid margins.

Statistically significant between-group differences in IOP were reported in the North American study from week 1 through week 16. By the week 20 visit, these differences had resolved [15]. At biomicroscopy and ophthalmoscopy in study 1, a one-grade increase in conjunctival hyperemia observed in 9.2 % of the subjects in the bimatoprost group was reported as an AE in 4.6 % of the cases (all cases rated as mild in severity). The rate of conjunctival hyperemia reported at biomicroscopy was consistent with that previously reported in a mostly Caucasian cohort from a prior study [15].

Study 2 enrolled subjects with chemotherapy-induced hypotrichosis of the eyelashes. These subjects showed greater improvements in eyelash growth later in the course of bimatoprost treatment than the idiopathic cohort in study 1. In addition, the vehicle group showed greater improvement in eyelash growth than the vehicle group in the idiopathic population. The observations in the vehicle-treated subjects may reflect natural hair-growth recovery after chemotherapy.

A limitation of these studies was their relatively short duration. Nevertheless, both studies showed statistically significant improvements in efficacy end points as well as a good safety profile. As noted earlier, the long-term safety of bimatoprost 0.03 % for glaucoma and ocular hypertension has been established. Moreover, a good safety profile has been demonstrated in a 1-year, phase 3 study of bimatoprost 0.03 % in subjects with hypotrichosis of the eyelashes (Clinicaltrials.gov identifier NCT00907426; results to be reported separately).

In summary, the current studies demonstrated that bimatoprost 0.03 % applied topically to the upper eyelid margins is effective in producing longer, thicker, darker, and more prominent eyelashes in Japanese subjects who have hypotrichosis of the eyelashes, with a favorable safety profile supported by ophthalmic examination.

## Study Sites

### Study 1

Study 1 included the following researchers and study sites: Takafumi Eto, MD (Tokyo Teishin Hospital Chiyoda-ku, Tokyo, Japan), Kiyonori Harii, MD (Kyorin University Hospital Mitaka-shi, Tokyo, Japan), Tomoko Hasunuma, MD (Kitasato Institute Hospital Biomedical Research Center Minato-Ku, Tokyo, Japan), Shinichi Hirabayashi, MD (Teikyo University Hospital Itabashi-ku, Tokyo, Japan), Atsuyuki Igarashi, MD (NTT Medical Center Shinagawa-Ku, Tokyo, Japan), Yuzo Komuro, MD (Juntendo University Urayasu Hospital Urayasu-shi, Tiba [Chiba], Japan), Ryoji Tsuboi, MD (Tokyo Medical University Hospital Shinjuku-Ku, Tokyo, Japan), and Rie Yamashita, MD (Shonan Kamakura General Hospital Kamakura-shi, Kanagawa, Japan).

### Study 2

Study 2 included the following researchers and study sites: Shinichi Hirabayashi, MD (Teikyo University Hospital Itabashi-ku, Tokyo, Japan), Yuzo Komuro, MD (Juntendo University Urayasu Hospital Urayasu-shi, Tiba [Chiba], Japan), Masujiro Makita, MD (The Cancer Institute Hospital of JFCR Koto-ku, Tokyo, Japan), Masaru Morita, MD (Fujieda Municipal General Hospital Fujieda, Sizuoka [Shizuoka], Japan), Masahiro Nakagawa, MD (Shizuoka Cancer Center Sunto-gun, Sizuoka [Shizuoka], Japan), Minoru Sakuraba, MD (National Cancer Center Hospital East Kashiwa-shi, Tokyo, Japan), Yuri Tokunaga, MD (Hamamatsu Medical Center Hamamatsu, Sizuoka [Shizuoka], Japan), Junji Washizu, MD (JA Shizuoka Kohseiren Enshu Hospital Hamamatsu, Sizuoka [Shizuoka], Japan, and Rie Yamashita, MD (Shonan Kamakura General Hospital Kamakura-shi, Kanagawa, Japan).
